# Mothers Who Accompany a Child to Their Death: Starting Again Without Ever Forgetting

**DOI:** 10.3390/nursrep15010015

**Published:** 2025-01-09

**Authors:** Maria Eduarda Correia, Maria Teresa Magão, Maria Antónia Rebelo Botelho

**Affiliations:** 1 Department of Nursing, Higher School of Health, Polytechnic Institute of Portalegre, 7300-555 Portalegre, Portugal; 2 Nursing Research, Innovation and Development Centre of Lisbon (CIDNUR), 1990-096 Lisbon, Portugal; mtmagao@esel.pt; 3 Research Center on Health and Social Sciences (CARE), Polytechnic Institute of Portalegre, 7300-555 Portalegre, Portugal; 4 Nursing School of Lisbon, 1990-096 Lisbon, Portugal; rbotelho@esel.pt

**Keywords:** parents, children, palliative care, phenomenology, nursing

## Abstract

**Background/Objectives:** Parents who accompany their children with a complex chronic illness until their death experience a unique situation, with vulnerabilities, specific needs and enormous suffering. The aim of the study was to describe the lived experience of parents who accompanied their children with a complex chronic illness until their death, in a paediatric palliative care setting. **Methods**: We opted for a qualitative methodology, with a descriptive phenomenological orientation. Phenomenological interviews were carried out with nine intentionally selected mothers, with the support of a paediatric palliative care hospital team. The procedural phases of van Kaam’s method, modified by Moustakas, were used to analyse the data. **Results:** An understanding of the essential structure of the phenomenon is revealed in a description made up of three essential themes: ‘facing the harbinger of illness’; ‘living (together) with a sick child’; and ‘starting again without ever forgetting: living with an absent child’, the latter being the subject of this article. **Conclusions:** The participants attribute a self-transforming meaning to their lived experience of accompanying their children. Nurses will be able to access the lived experience of these mothers and improve their intervention in the process of their children’s illness, as well as in their bereavement process. There are also contributions to research and teaching in palliative care in the area of child and paediatric health.

## 1. Introduction

Technological progress and scientific research in recent decades have led to the development of health care, changing the profile of patients and diseases, and in paediatrics, it has brought undeniable progress, making care for children with complex chronic disease (CCD) increasingly complex, developed and differentiated [1,2,3,4,5,6,7].

A CCD is any life-limiting illness lasting at least 12 months (unless death occurs), severely affecting one or more organs and for which there is no hope or possibility of a cure. For this reason, these children require differentiated, prolonged and interdisciplinary paediatric healthcare, with the possibility of one or more periods of hospitalisation in a tertiary-level hospital [8,9].

These children need differentiated, permanent and expensive healthcare, are often dependent on medical devices and technology to survive and/or avoid complications, usually provided by their parents, and are supported by healthcare professionals, who respect and recognise their role as carers, providing them with the necessary knowledge and training, valuing their concerns about their sick child’s well-being, and treating them as a unique being and not as a disease [10,11,12,13,14].

Parents who accompany their children with CCD conditions on a daily basis, often dependent on technology, in a hospital, community or home setting, are confronted daily with countless difficulties, conflicts, loss and suffering, but demonstrate incredible strength through a multitude of ways to make their child ‘happy’, regardless of their health condition [10,11,12,13,14].

Mothers are the main carers, taking on this mission, caring at home or during periods of hospitalisation, in partnership with health professionals with family support, living according to their child’s needs which are their priority, and putting other family or professional responsibilities on the back burner [15,16]. The father continues to take on the role of pursuing his professional activity and is the family’s main financial support [15,16,17].

Parents who accompany and care for their children with CCD are constantly dealing with the disease, facing complex and difficult situations. They often find themselves with many doubts and few certainties, trying to reconcile the surprises and disappointments associated with the hope and hopelessness of the child’s future, while at the same time experiencing a duality of feelings, not only of managing their own emotional state but also of being the support for their sick child, a fact that often extends to the rest of the family [6,10,11,12,13,14,15,16,17,18,19].

The philosophy of paediatric palliative care (PPC) is based on holistic and proactive care centred on the sick child, parents and family, through humanised technical intervention seeking maximum comfort and quality of life, promoting their autonomy and dignity [2,3,4,5,6,7,20]. PPC is not and does not act in opposition to curative therapy. The integration and interconnection of the two models, curative and palliative, can provide a better quality of life for children and families in need of differentiated, complex and interdisciplinary healthcare [21].

PPC is an inter- and multidisciplinary model of intervention involving the active and total assistance provided to children, in the dimensions of their body, mind and spirit, as well as the support offered to their family, which is considered the unit of care [21].

Knowing the uniqueness of the lived experience of parents who accompanied their children with CCD until their death, in a PPC context, is essential because caring in paediatrics is caring for the child and also caring for the parents [20,21,22], making it necessary to know their needs, difficulties and concerns [23,24,25,26].

Human experience is a complex entity, dynamic and continually in movement [27,28,29].

The phenomenological meaning of ‘lived experience’ represents the original way in which human beings exist in the world as beings in their own right and implies that the essence of this experience lies precisely in its ‘lived’ character, and not in what is felt or experienced in the passage of time, but what is significantly detached and preserved from that passage of time [26,27,28]. The phenomenon of experience can be seen as something that happens to, overwhelms or affects the person, and can be understood more actively as an act of consciousness in appropriating some meaning from some aspect of the world [29,30].

Health professionals who care for these children may be able to support parents in finding hopeful thinking while looking after their children and lay the foundations for a healthier grieving process, in which the child’s memory is valued and not a source of despair or suffering [15,17,19].

Jean Watson refers to the need to study and investigate the area of human experience in health and to develop methods that preserve the human context and allow for the advancement of knowledge about the world of lived experience [31].

The literature review shows an increase in studies in the context of PPC. However, little is known about the experience of parents during their child’s illness [32]. The knowledge produced through research centred on uncovering the phenomenon of parents accompanying their children with CCD until their death, in a PPC context, will make it possible to access the narrative of the lived experience.

It is therefore important to gain access to their lived experience through studies that allow us to uncover and understand the phenomenon of these parents’ experiences of accompanying their children throughout the illness process [10,11,12,13,32,33,34,35,36,37,38,39].

Considering the need described above, a study was carried out with the aim of describing the lived experience of parents who accompanied their children with CCD until their death, in a PPC context.

## 2. Materials and Methods

In view of the problems presented, this study is part of a qualitative paradigm, using van Kaam’s (1959) descriptive phenomenology and van Kaam’s data analysis method modified by Moustakas (1994) [28,40]. Phenomenology means the study of phenomena, the study of what appears in consciousness, promoting access to the pre-reflected world as we experience it. The pre-reflected is the original experience, the immediate contact with the world before it becomes conscious to the subject [28,31,32,41]. But phenomenology is also a methodology for investigating phenomena as they are experienced in each person’s consciousness [28,31,32,41]. Phenomena related to nursing can be explored and analysed using phenomenological methods, which aim to describe the lived experience in a reliable way [28,31,32,41]. A phenomenological analysis of human experience in health and illness can provide a description of the meaning of lived experience. As such, the experiences can lead to an increased understanding of human behaviour in health and illness and the means to explore the human process of caring [33].

### 2.1. Participants

#### 2.1.1. Inclusion and Exclusion Criteria

The participants were nine mothers of children of paediatric age (from 0 to 18 years old) who had been involved in the process of accompanying their children with CCD, in contexts of hospitalisation and outpatient and/or home care by PPC units and/or teams. Participants were selected intentionally and the following inclusion criteria were defined: age 18 or over; having understood the aim of the study; having the verbal communication skills to be able to describe the phenomenon under study; wishing to take part in the study; living in mainland Portugal; the death of their child having occurred in a period of 12 months or more; and being the mother and/or father of a child (0 to 18 years of age) with CCD whom they accompanied in a PPC. The exclusion criteria were not being able to speak Portuguese and not living in mainland Portugal.

#### 2.1.2. Participant Selection Process

The participants were selected as follows:

The first contact was made by telephone by a member of a PPC Intra-Hospital Team, after learning about the inclusion and exclusion criteria for participants in the research, as well as the monitoring carried out during the child’s illness and the parents’ bereavement process. Mothers were intentionally nominated by a PPC Intra-Hospital Team professional to take part in the research. After the mothers agreed to take part in the research, the study researcher learnt about the participants and their contact details and made a second telephone contact. In a third contact, the participant, after confirming her intention to accept the invitation to take part in the research, set the date and place of the interview.

Throughout the selection process, the dates of the child’s birthday, birth and death were taken into account in all the contacts made with the participants, as it is described in the literature that both dates reinforce the memories of the children with the bereaved parents and can generate moments of greater emotion and sensitivity [42].

### 2.2. Data Collection

Data collection through the phenomenological interview is characterised by being unstructured, in-depth and open, which allows access to the world of the other, thus constituting an excellent source of data on the person’s lived experience and that lived situation and/or context [29,31,32,43,44]. At the start of the interview, the objectives of the research and all the rights of the participants were recalled and validated and any questions/doubts that had not already been clarified in previous contacts were clarified. The interview was conducted on the basis of a guiding question, in order to place the participant in the interview topic.

It was also important that the location of the interview provided a relaxed atmosphere and that the participants felt comfortable throughout.

The interviews took place face-to-face and individually between 5 August 2021 and 13 April 2022, at a location chosen by the participants. Of the nine participants, seven chose their homes and the rest chose municipal gardens in the cities where they lived. The interviews lasted an average of 1.32 h, and the maximum time elapsed between the death of the participants’ child and the interview was an average of 35 months.

The diary was also an important tool for the notes (the researcher’s sensory perceptions, non-verbal language and ideas) that resulted from the researcher’s observations and reflections during the interviews, which helped in analysing and discussing the data [40].

During or after the interviews, there were no episodes identified by the researcher and/or the participants of discomfort and/or emotional disturbance in which it was necessary to interrupt the interview, provide emotional support and refer for a bereavement consultation (specialised support) by the PPC Intra-Hospital Team.

### 2.3. Data Analysis

After the interviews had been carried out, the researcher transcribed them one by one, in their entirety, without interpreting or altering the meaning, and literally, respecting the personal way in which each participant appropriated and made use of language as a communication tool. The participants were coded. In order to maintain the participants’ anonymity and the confidentiality of the data, the researcher eliminated all data that could allow the participants and their children to be identified, so she used fictitious names for the coding and designation of both.

The data were processed and analysed by the researcher, with the collaboration of the supervisors and researchers from the Fundamental Research Group of the Lisbon Nursing Research, Innovation and Development Centre (CIDNUR), with the aim of understanding the nature of human health phenomena, with lived experience as the focus of nursing care, in the context of collaborative analysis [30].

In order to access the world of the lived experience of the mothers who accompanied their children to death in the context of the PPC, the data were analysed from the perspective of descriptive phenomenology, using the seven procedural stages of the van Kaam method modified by Moustakas [28,40], as follows:

(1) Listing and Preliminary Grouping: Horizontalisation—In order to understand the overall meaning of the nine narratives, all the texts were read individually and randomly. This prevents the longer narratives with more descriptions of the phenomenon from overlapping with the others, thus having a greater influence on the final result of the data analysis. Once the researcher had carried out this initial reading, she drew up a list of all the participants’ descriptive expressions, which she considered to be significant for understanding the essence of the phenomenon.
(2) Reduction and Elimination: Determining the invariant constituents—The researcher repeated the individual reading and analysis of the narratives, recognising expressions and/or phrases that described the invariant constituent elements of the experience. The invariant constituents are abstract statements that tend towards a theme, appearing explicitly or implicitly in each of the narratives, and which need to be reconcilable with the descriptions visible in their essence [28,40]. In this process, repeated expressions and/or phrases considered irrelevant to understanding the phenomenon under study were also identified and eliminated.
(3) Categorisation and Thematisation of the Invariant Constituents—The researcher carried out the data analysis process, grouping and classifying the expressions and/or phrases she considered to be invariant constituent elements of the experience. At this stage, themes were created that constituted the essence of the phenomenon, which made it possible to draw up the themes of the phenomenon.
(4) Final Identification of the Invariant Constituents and Themes by the Application: Validation—In this stage, the researcher verified the data analysis carried out in the previous stages by re-reading the participants’ narratives. This stage was finalised when the invariant constituents proved to have meaning for the experience, both in the transcriptions of the participants’ expressions and/or phrases and in the totality of the phenomenon.
(5) Building an Individual Textural Description—In this stage, the researcher sought to build an individual textural description using the ‘verbatim’ expressions and/or phrases transcribed from the participants’ narratives. The construction of the individual textural description reflects the origin of the phenomenon and is therefore more objective than the invariant constituents and themes analysed in Stage 4, as it reproduces the nature of the phenomenon.
(6) Construction of the Individual Structural Description—In this stage, an abstract individual structural description of the experience was constructed, and for this, the researcher drew on her personal and professional experience. The purpose of the analysis was to find the necessary constituents of the experience, initially in text form and then structurally. This process is dynamic and requires ‘going back’ to the participants’ narratives. It was also important at this stage for the researcher to write down the descriptive hypotheses of the phenomenon, as this process was intended to contribute to the rigour of the data analysis.
(7) Composite Description: Construction Of Structural–Textural Description—In the last stage, the researcher brought together the meanings of the lived experience in the so-called structural–textural description, adjusting the individual textural description with the individual structural description. The final analysis of the data was presented through a composite description together with the elaboration of the geometric figure, both of which represent the totality of the phenomenon, where the communalities are highlighted and the individual descriptions are attenuated, in the essence of the structure of the experience of the phenomenon.

The various stages and procedures of the method take place simultaneously without any time limits, with no identifiable beginning or end [28,40].

### 2.4. Rigour and Reflexivity

Phenomenology as a research method is centred on lived experience and not on the factual accuracy of a story [29,41,45,46].

It is essential to use strategies that ensure both the appropriate use of the phenomenological method and the rigour of its results. The researcher must therefore establish and use appropriate rigour strategies that are duly framed in the methodological process of the research [29,41,45,46].

In the course of methodological procedures, adequacy and rigour are essential to the credibility, transferability, dependence and confirmability of qualitative research [29,45,46]. These concepts are associated with the rigour strategies followed in qualitative studies. If they are not used or used inappropriately, they become a threat to the credibility of the study’s results [45,46].

*Credibility* refers to the researcher’s ability to analyse and the quality of the information gathered [45,46]. This research involves nine participants who were nominated by the PPC Intra-Hospital Team and who accepted the invitation to take part in the research. All the interviews and their transcriptions were carried out individually by the researcher. After the last interviews had been carried out, nothing else seemed to emerge to describe the phenomenon under study.

The *transferability* of the data is associated with the participants, and this process is facilitated through a purposive sample of participants [45,46]. The fact that the participants were selected intentionally (taking into account previously established inclusion and exclusion criteria) increased the chances of finding relevant information about the phenomenon under study (accompanying children with CCD until their death in a PPC context). A schematic representation of the phenomenon under study was also drawn up, i.e., the structural organisation of the essential elements of the phenomenon, in order to make it easier to understand [45,46].

*Dependence* requires the researcher to draw up detailed documentation of the entire research process, the methodological decisions made and the assessment of the research results [45]. Therefore, one of the strategies is to ensure that, given the detailed documentation of the research process and the methodological decisions made, other researchers are able to follow the process and reach similar conclusions, taking into account the data collected and the context [45,46]. In the methodological process, the decisions were shared by both supervisors and experienced researchers.

Finally, *confirmability* goes hand in hand with objectivity, that is, the extent to which the results of the study are the product of the research focus and not an arbitrary interpretation by the researchers themselves [45,46]. An appropriate route should allow an external, properly trained researcher to determine whether descriptions, interpretations and recommendations can be traced back to their source.

To be considered valid, a phenomenological study must take into account methodological congruence (rigorous and appropriate procedures) and experiential concerns that provide insight in terms of plausibility and clarification of a specific method [29,41].

### 2.5. Ethical Considerations

A favourable opinion was obtained from the Hospital Centre’s Ethics Committee (no. 052/CES).

Each participant signed the consent form together with the researcher on a separate form and in duplicate, with one copy being kept by each of them. The anonymity and confidentiality of its content were guaranteed, and its use was restricted solely and exclusively to the research in question. There was no monetary payment or material compensation for taking part in the research.

Authorisation was requested for the interview to be audio-recorded and the participants were informed that they could interrupt and leave the interview at any time, if they wished, without any harm to themselves [29,32,41,44].

In a health/illness context, research often involves studying a population made up of individuals who are temporarily or permanently frail and/or vulnerable; for this reason, they must be given extra care, given the duty to protect those who are most frail and vulnerable.

The principal investigator reinforced her training in order to be qualified to provide emotional support to the participants during data collection if they needed it.

Given the ethical challenges that the vulnerability of the research participants could raise, they were selected by a member of the PPC Intra-Hospital Team and it was ensured that the bereaved participants who agreed to take part would have access to the professionals of the PPC Intra-Hospital Team, so that they could resort to emotional and psychological support if they needed it. It was therefore ensured that the bereaved mothers who agreed to take part in the study would have access to the professionals of the PPC Intra-Hospital Team so that they could call on this additional support if they needed it.

## 3. Results

Understanding the essential structure of the phenomenon is revealed in a composite description of the lived experience of the mothers who accompanied their children until their death in the context of PPC, which involves three essential themes: ‘facing up to the harbinger of illness’; ‘living (together) with a sick child’; and ‘starting again without ever forgetting: living with an absent child’ (Figure S1: Schematic representation of the composite description of the accompaniment phenomenon that emerges from the mothers’ lived experience).

The aim of this article is to present the theme ‘starting again without ever forgetting: living with an absent child’.

### 3.1. Characterisation of the Participants

All the participants are female, of Portuguese nationality, living in mainland Portugal and are characterised by being mothers and also carers of their children, accompanying them throughout their CCD process, in a PPC context. The participants’ ages at the time of the interview ranged from 32 to 46, with an average of 39.1 years. Regarding their marital status and family situation, eight participants remained married/married in fact and one participant was divorced. Most of the participants had compulsory schooling (Table 1).

The participants’ children, who were followed through the CCD process in a CPP context, were five males and four females, all of Portuguese nationality and residents of mainland Portugal. Their ages at the time of death ranged from 3 months to 16 years. As for the children’s clinical diagnoses, two children had cerebral paralysis, one had an oncological disease (a malignant tumour of the cerebellum), three children had congenital heart disease, one child had severe neuromuscular disease (spinal muscular atrophy type I), and two had clinical diagnoses of irreversible diseases with severe disability that led to health complications that resulted in premature death (hypoxic–ischaemic encephalopathy and oesophageal atresia). Regarding the place of death of the children, two died at home with their immediate family and the remaining seven died in hospital (Table 2).

### 3.2. Starting Again Without Ever Forgetting—Living with an Absent Child

To accompany a child until death is to fulfil a great and transforming mission. Mothers attribute meaning to this transformation, in the sense of changing and revaluing life. What used to not be the focus of their attention and thoughts, they now attribute another value to, such as the sun and the way it shines, and the beauty of the simple things in nature. On the other hand, their monetary status and material value are pushed into the background. This is how the participants Madalena, Margarida, Manuela, Marta, Mónica and Matilde present it in their narrative:

Madalena (P2)—‘It was an experience that I never thought I would have, but I think I was destined to go through this, and to make me even stronger, and with the help of him who looks after me, of God, I became a better person, and that was my experience’.

Margarida (P4)—‘I’m not the same person today, I’m not the same Margarida, I’m a much better person, I’m much more tolerant, I’m a much more understanding person (...) and I would have lived it all over again’.

Manuela (P5)—‘Everything was a learning experience for me and I learnt and I always tried to make an effort to do everything that was necessary for my son, I was going to be there for everything and learn everything that was necessary, in the meantime I went from being a mother to a nurse, a doctor, a psychologist, all this in quotes, so I also enriched myself as a person, I also learnt from my son’s illness, I became a different person’.

Marta (P6)—‘After going through everything I’ve been through, then everything else has a different value, I value certain things differently (...) having the sun beating down on my face has a different value (...) what changed me was valuing the things that are part of life, of everyday life, that you didn’t think about before’.

Mónica (P7)—‘And also after this experience of illness with my daughter, I’ve learnt to be different (...) and I also know that I’m not the same, but I’ve learnt to value the situation of families with very sick children, the world of the hospital and maybe my daughter has given me the opportunity to become a different person because she’s been through all this’.

Matilde (P9)—‘It’s been an enriching experience, the greatest life story I’ve had so far (...) all the lessons he’s given me, all the tools he’s given me, if I’m a better person I owe him and I’ll never forget my son (...) you know, parents who have lost children, we know how to value the little things’.

Bereaved mothers feel an emptiness and an intention to rediscover and extract a new meaning from life because, after dedicating themselves to their sick child, there is a need to continue their duty of care, maintaining procedures, habits and routines. Living with the absent child is the result of continuing their duty of care, now not of his body, but of his soul, his memory, his legacy, as the participants Madalena and Maria narrate, respectively:

Madalena (P2)—‘it was going to the cemetery, taking care of him, washing the stone, putting flowers on his grave, I feel the need to look after him, to continue looking after him as if he were still with me, to be his mother, to continue being his mother, I have another daughter, but I need to continue, I have this need, to feel this need, to take care of him, to give him importance, to keep him alive inside me, it’s as if this feeling is fighting against his death, life against death and not forgetting what he would need if he were alive, his needs, his demands as a child’.

Maria (P3)—‘At the end of the day, I feel that he’s physically dead, but I have to carry on being with him, looking after him. Now I don’t look after his body, but his soul, his memory, that’s very important to me, there’s not a day that goes by that I don’t remember my son and everything he needed from me that I had to do for him to live, as I said, I lived for him and always with him (...) accompanying my son is forever, it didn’t end with his death’.

Despite the death of their children, the participating mothers do not stop being mothers. That is why they maintain their relationship with their child through conversation, prayer and even written letters, because there is a fear of forgetting their child, their particularities, and their uniqueness, not only for themselves, but also for others. Also, on festive dates, celebrations, birthdays and important events (family and/or other), they make a point of remembering their absent son, never giving way to forgetfulness, but rather focusing onmemories and recollections. Participants Margarida, Marta and Mónica describe this in their narratives:

Margarida (P4)—‘We cremate our daughter, our daughter is in our room, I wake up, I look at my daughter, I tell her I love her, just like I told her every day while she was alive and then I go on with my life (...) my journey with my daughter has come to an end here, here in life, but I still write letters to my daughter, telling her things about her sister’.

Marta (P6)—‘I haven’t stopped being my daughter’s mother, I’m still her mother, you don’t stop having a child because my heart still hurts, you never stop being a mother, regardless of the age at which you lose a child, you never stop being a mother, I felt that it wasn’t just the physical loss I had with my daughter’s death, it was more than that’.

Mónica (P7)—‘My daughter is always present in our lives, I always talk about her with my husband and even with other people at work (...) we always remember her, we always remember her, she’s everywhere in our house, we always go to the cemetery on Saturdays to decorate it, if we can’t go on Saturday for some reason we go on another day, but every week we go there, her room is still there as it was when she was alive, she’s always remembered, especially on her birthday, but it’s hard because she is not forgetter and I’ll never forget her, just yesterday at my in-laws’ house they were talking about her’.

The participants Madalena and Maria believe that after death, their son became immortal, a star or an angel in heaven who now also fulfils a mission to protect all those who live on Earth and for whom his life story is a source of inspiration:

Madalena (P2)—‘his soul has gone to heaven because he’s a little angel, but I was ready to talk and I prayed a lot, so that he in heaven would look after us, all of us in the family, as if he were now our more than divine protection’.

Maria (P3)—‘the priest said that my son was an angel and a saint that the earth had won, and I don’t know to what extent we’re not really witnessing this, if this isn’t really true, because I know that there are people who clung to my son, to my Pedro (...) he’s up there in heaven helping God, he’s helping us all, because I always ask him to help us all because all those who have passed through Pedro’s life he doesn’t forget anyone, and he’s helping’.

Death imposes physical separation, accompanied by the pain of loss that is untouchable and permanent, but it is the longing that lasts over time that is the hardest feeling to fight. To start again without ever forgetting is also to hope that you can conquer the ability to get back on your feet, to rebuild, and that sometimes means planning for a new motherhood in the near future.

Marília (P1)—‘(...) it’s a great pain, the longing is great, the memories, the longing for her smile, her hug, these things..’.

Maria (P3)—‘People often say that I talk about my son with a very big smile, but I talk about him because he was a very happy boy and he was very loved, very wanted, very desired, he was loved everywhere and time passes and the longing grows more and more and no one can solve that’.

Margarida (P4)—‘I am proof that there is hope after death, after the loss of a child, because this daughter of mine is now healthy and a perfectly normal girl and she helps us a lot in our process’.

Manuela (P5)—‘I had to get on with my life and I found a house and also left the town where I lived (...) and when I first entered this house I didn’t bring anything but my body clothes, I had nothing and I said I’m going to get on with it whatever it takes and I started looking for work (...) my reaction after the death of my son and my grieving process was always to move on’.

Matilde (P9)—‘Rui is life, he’s the life that has given me more joy in my days, he’s my future, that future that had been put on hold with Henrique, but he hasn’t replaced it, he’s added to my life, just as my first son was a lesson in my life, and still is today’.

## 4. Discussion

The death of a child defies the natural order, representing an irreparable and disturbing loss, leading to profound consequences both on an individual level and on the level of the family where it occurs, with consequent suffering on the part of the parents who experience something they never expected to be confronted with, such as the death of someone they love, someone who is the centre of their universe [10,12,47,48,49].

After the death, with respect to the theme of ‘*starting again without ever forgetting: living with an absent child*’, bereaved mothers continue to accompany and care for their child, but now preserve their memories, their legacy and their presence, for themselves and for others, nurturing hope and gaining the ability to rebuild themselves and make sense of the transformation of their experience, and rebuilding themselves in search of another new meaning of life [50,51].

The mothers feel that accompanying their sick child was a lesson they will never forget, despite all the suffering, pain and sadness they experienced. But if they had the choice, they would repeat the experience without any hesitation because they believe that they were chosen to live this story and that through their experience they have become better people, more tolerant and more understanding, with a greater capacity to forgive others, valuing life more in the present and less in the future [36,52].

The results of a qualitative study carried out in Brazil and published in 2021 also show that mothers’ experience of bereavement is characterised by a unique process of transformation and adaptation. The main strategies found by bereaved mothers were support from family and friends; sharing feelings and experiences with other bereaved mothers; returning to work; perpetuating the memory and keeping objects of the deceased child; a new pregnancy; changing spaces in the house; spirituality; and realising their own dreams and those of the deceased child [42].

According to previous studies, after the death of a child, bereaved mothers feel the need to continue caring for their child as if they were still alive. There is a need to remain close to their child, which is why they make daily and/or frequent trips to the cemetery, or to the place of worship and homage in their own home with objects, photos and toys so that everyone feels their presence and they are never forgotten. After death, the mission of being a mother remains, so that the child stays alive, in the family [36,50,51,52,53,54].

After death comes the need to continue caring for the sick child, as if they were still alive, to continue their existence and to continue being a mother, and the need to remain close to the child arises, which is why they make daily and/or frequent trips to the cemetery, or to the place of worship, and pay homage in their own home with objects, photos and toys so that the parents and even others feel their presence and that they are never forgotten, continuing to be part of the family, and that their place is not taken by anything or anyone else [36,50,51,52,53,54].

However, following the transformation they undergo, mothers embark on a process of reframing and redirecting their lives, embracing a new way of being in the world. The participating mothers express and attribute meaning to the transformation they have undergone as a result of the experience of accompanying their children with CCD until their death. This process of transformation leads them to identify with the revalorisation of the self. The pain of loss is a constant, and nostalgia is arguably the most challenging emotion to overcome. However, it is a transient phenomenon, as death signifies a physical separation, yet the bond and sense of belonging are not disrupted by it. Consequently, the experience of caring for a child with CCD is indelibly etched in the mother’s memory. However, after undergoing a transformative experience, mothers tend to reframe and redirect their understanding of themselves and their place in the world, prioritising self-care [42,50,51,52,53,54,55,56].

The expression ‘*self-care*’ is used to represent a complex notion to designate a series of attitudes linked to the circumstance of taking care of oneself and worrying about oneself [57,58].

On the other hand, other studies also consider that ‘self-care’ is related to the perception that people should take care of themselves, implying a double meaning in which, as well as referring to the inherent care one should have for oneself, it has also come to encompass the care one should have for *others* [57,58,59].

In this sense, we can mention through the narratives of the bereaved mothers that *self-care* is a new attitude linked to the exercise of being and being in the world, a new way of looking at things, of relating to others and to oneself; of acting from oneself to oneself; and of modifying oneself, purifying oneself, transforming oneself and transfiguring oneself [57,58,59].

This study shows us how the human phenomenon—accompanying a child with CCD until death—as an individual process involving the experience of enormous suffering is also one of transformation, through the capacity for reconstruction, of a new being-in-the-world sentiment, crossing into the future through a new meaning in life [55,56,57].

## 5. Conclusions

The lived experience of accompanying a child with CCD until their death in a PPC context is a phenomenon of enormous complexity and uniqueness and illustrates the enormous suffering, not only in the process of illness, but for their entire existence. For these mothers, accompaniment is a duty, feeling the responsibility of fulfilling a mission to which they were destined, so it is unquestionable, and there is no chance of it being refused or interrupted. The experience of accompanying a child with CCD until death, in a PPC context, conditions the transformation and reconstruction of a new being, in search of another sense of being in the world experienced with longing, but in the continuous mission of caring.

The data point to possible implications for nursing in terms of clinical practice, research and teaching.

Considering possible implications for clinical practice, the data from the study could lead to humanised care, based on an understanding of what mothers of children with CCD experience. The data also point to support for grieving parents and the rest of the family after the death of the child, in order to alleviate the suffering and sadness of the significant loss and avoid prolonged and/or pathological forms of grief [39,47,50,60].

An effective health gain could be the reorganisation and allocation of human resources in PPC teams to enable health professionals to provide holistic support to bereaved parents and relatives, in a listening and caring environment, taking into account existing recommendations [55]. From a research perspective, the data could inform the design of future qualitative studies exploring the lived experience of parents accompanying their children with CCD in a PPC context. It is also important to consider the potential for further research that incorporates diverse cultural and ethnic perspectives. Given the limited number of studies employing van Kaam’s methodological approach [28,61], it is considered relevant to use it to guide future qualitative studies in the area [37].

In terms of teaching the subject, the results point to the need for nursing training in the development of skills in the field of paediatric palliative care, allowing for sustained planning through international and national recommendations to facilitate and stimulate the effective and relevant integration of paediatric palliative care content at the different levels of training.

This research has made it possible to understand the phenomenon, and it is therefore important that the data can be disseminated so that nurses, as well as other health professionals, can have access to the knowledge produced about the experience of accompanying a child in the context of PPC, which can improve health care for the child and their family.

## Figures and Tables

**Table 1:** Sociodemographic characteristics of the participants.

Participant	Age	Marital Status	Education
Marília (P1)	46	married	12th year
Madalena (P2)	45	married	4th year
Maria (P3)	46	married	12th year
Margarida (P4)	38	married	professional course
Manuela (P5)	34	divorced	secondary education
Marta (P6)	39	married	12th year
Mónica (P7)	40	married	professional course
Mafalda (P8)	32	married	professional course
Matilde (P9)	32	civil partnership	12th year

**Table 2:** Characterisation of children with complex chronic disease.

Participant’s Child	Diagnostic	Age at Death	Place of Death
Palmira, daughter of P1	cerebral paralysis	12 years	hospital: palliative care
Sebastian, son of P2	tetralogy of Fallot	15 months	hospital: intensive care
Pedro, son of P3	West syndrome	16 years	home
Patricia, daughter of P4	congenital heart disease	1 year	hospital: intensive care
Paulo, son of P5	malignant tumour	4 years	hospital: palliative care
Pureza, daughter of P6	type I spinal muscular atrophy	13 months	home
Pilar, daughter of P7	hypoxic–ischaemic encephalopathy	6 months	hospital: palliative care
Piedade, daughter of P8	hypertrophic cardiomyopathy	3 months	hospital
Henrique, son of P9	oesophageal atresia	9 years	hospital: palliative care

## Data Availability

The data that support the findings of this study are available from the corresponding author upon reasonable request.

## Supplementary Materials

The following supporting information can be downloaded at: https://www.mdpi.com/article/10.3390/nursrep15010015/s1, Figure S1: Schematic representation of the composite description of the accompaniment phenomenon that emerges from the mothers’ lived experience).

## Public Involvement Statement

There was no public involvement in any aspect of this research.

## Guidelines and Standards Statement

This manuscript was drafted in accordance with the Standards for Reporting Qualitative Research (SRQR).
