# Supplementary material for: Mothers Who Accompany a Child to Their Death: Starting Again Without Ever Forgetting
Source: Nurs Rep. 2025 Jan 9;15(1):15. doi: 10.3390/nursrep15010015 (PMC11767767; doi:10.3390/nursrep15010015)
Supplement: Supplementary file 1 [file nursrep-15-00015-s001.zip › nursrep-3354994-supplementary.pdf]

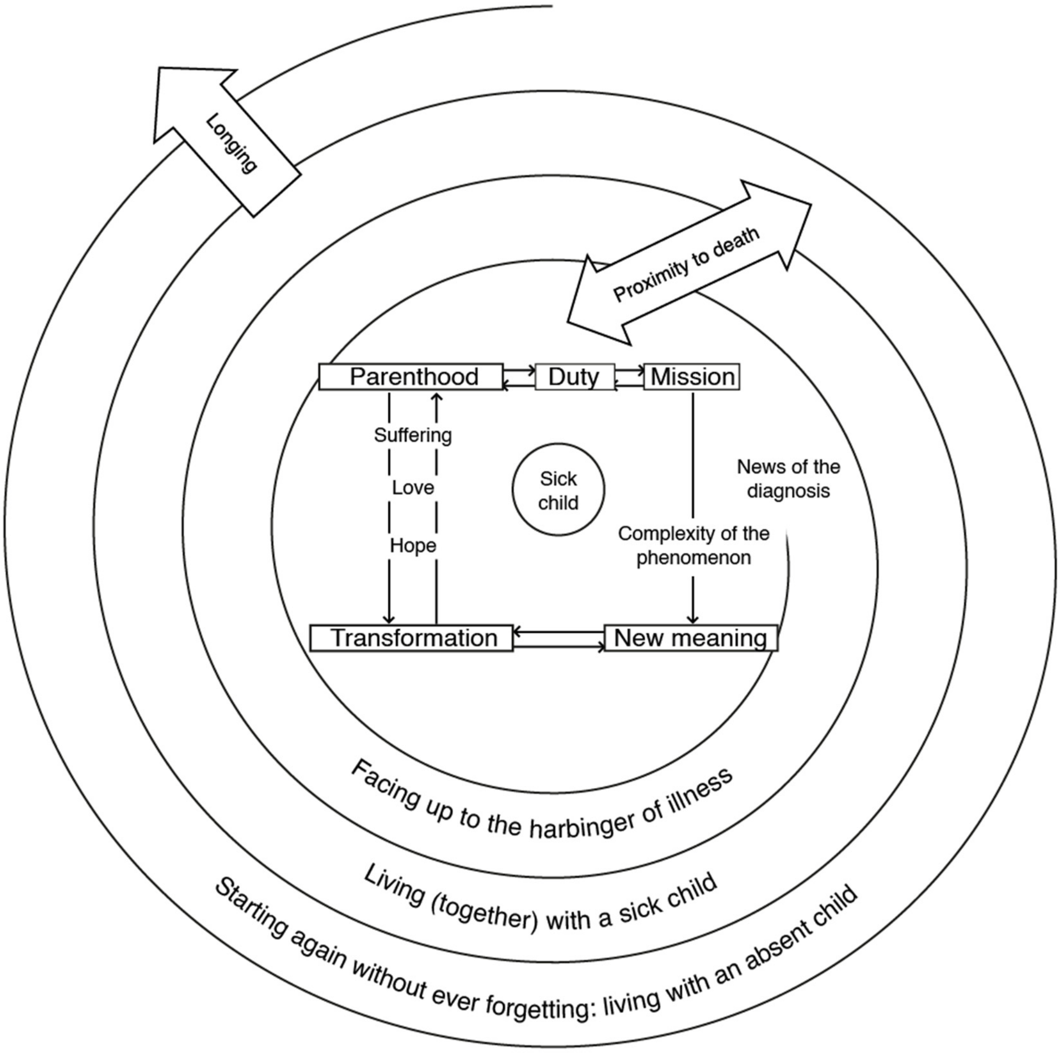

Figure S1: Schematic representation of the composite description of the accompaniment phenomenon that emerges from the mothers' lived experience).
